# Large multicenter validation of urine RNA profile for urothelial carcinoma detection and surveillance

**DOI:** 10.1172/JCI203094

**Published:** 2026-04-09

**Authors:** Kathleen E. Mach, Zachary Kornberg, Eugene Shkolyar, Jin Long, Timothy J. Lee, Vinh La, Ihna Yoo, Gabriela Rodriguez, Alan E. Thong, Kris B. Prado, Jay B. Shah, John T. Leppert, Eila C. Skinner, Joseph C. Liao

**Affiliations:** 1 Department of Urology, Stanford University School of Medicine, Stanford, California, USA.; 2 Veterans Affairs Palo Alto Health Care System, Palo Alto, California, USA.; 3 Department of Pediatrics, Stanford University School of Medicine, Stanford, California, USA.

**Keywords:** Clinical Research, Oncology, Biomarkers, Cancer, Urology

## Abstract

**BACKGROUND:**

Current diagnosis and surveillance of bladder cancer rely on cystoscopy, which is invasive and user dependent. The urine mRNA panel, uRNAp, measures expression of 3 genes for identification of bladder cancer. Here we report validation of uRNAp for patients undergoing initial workup for suspected bladder cancer and surveillance for bladder cancer.

**METHODS:**

Urine specimens were prospectively collected prior to cystoscopy at 2 health care systems from patients without (detection cohort) or with (surveillance cohort) a history of bladder cancer. RNA was isolated from urine sediment for RT-qPCR to determine roundabout guidance receptor 1, corticotropin releasing hormone, and insulin-like growth factor 2 expression and calculate the uRNAp bladder cancer probability score.

**RESULTS:**

In the detection cohort, 547 samples were collected from 529 patients. There were 123 new diagnoses of bladder cancer in the detection cohort, and uRNAp demonstrated 98% sensitivity and 51% specificity for identification of bladder cancer. In the surveillance cohort, 1,543 samples were collected from 447 patients with 286 recurrences. uRNAp demonstrated 94% overall sensitivity with 43% specificity and 99% sensitivity for high-grade recurrence. The receiver operating characteristic area under the curve was 0.92 in the detection and 0.81 in the surveillance cohort. uRNAp scores significantly increased with tumor size and grade.

**CONCLUSION:**

Prospective validation of uRNAp demonstrated a strong potential clinical utility as a noninvasive adjunct to cystoscopy for management of bladder cancer. uRNAp may be a useful triage tool to defer or expedite cystoscopy for patients undergoing detection or surveillance of bladder cancer.

**FUNDING:**

Department of Veterans Affairs BLR&D Merit Review I01 BX004962 to JCL.

## Abstract 2

<span>We developed a highly sensitive urine biomarker assay for detection and surveillance of bladder cancer with the potential to triage the need for invasive cystoscopy.</span>

## Graphical abstract

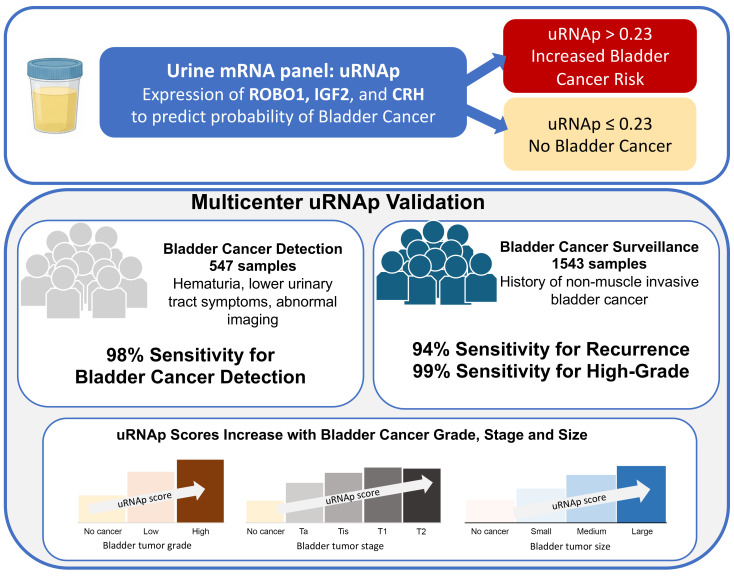

## Introduction

Urothelial carcinoma (UC) is the most common malignancy of the urinary tract, accounting for over 90% of the cancers of the bladder and upper urinary tract (1). Bladder cancer is the seventh most common cancer in the United States, with more than 80,000 new diagnoses and 16,000 deaths in 2024 (2). The incidence of bladder cancer is about 4 times higher in men compared with women (3, 4). Most patients with bladder cancer initially present with hematuria or lower urinary tract symptoms that indicate the need for radiographic imaging and direct visualization of the bladder and urethra with cystoscopy (5, 6).

Non-muscle-invasive bladder cancer (NMIBC) accounts for nearly 75% of cases at presentation and is primarily managed with transurethral resection of bladder tumor (TURBT) followed by intravesical therapies based on risk stratification (6, 7). For muscle-invasive bladder cancer, radical cystectomy with or without neoadjuvant chemotherapy is the first-line treatment (5). The risk of recurrent NMIBC varies greatly and can be stratified based on grade, stage, tumor size, multifocality, presence of carcinoma in situ (CIS), and recurrence interval (8, 9). With recurrence rates for bladder cancer of 15%–61% at 1 year and 31%–78% by 5 years, frequent surveillance after initial treatment is essential (8).

Initial detection and long-term surveillance of bladder cancer rely on cystoscopy in the clinic setting. If bladder tumors are found, TURBT is performed in the operating room for pathological diagnosis and local staging. Cystoscopy has a diagnostic accuracy of 70%–80% and may miss small papillary tumors and flat-appearing CIS (10). The need for frequent cystoscopic surveillance can negatively impact a patient’s quality of life and is burdensome in low-resource clinical settings (11, 12). Cystoscopies require in-person clinic visits as often as every 3 months, contributing to the high health care cost associated with bladder cancer (12). Additionally, most patients experience some level of anxiety and discomfort/pain associated with the procedure, and the majority of patients report at least 1 adverse event associated with cystoscopy, including hematuria, dysuria, and urinary tract infection (13, 14). Surveys of patients undergoing cystoscopy indicate they would be receptive to urine tests, with about 75% of surveillance patients indicating that they would be willing to accept a urine test with least 90% sensitivity and 60% of detection patients indicating a preference for a urine test instead of cystoscopy (13, 14).

Urinary biomarkers to identify patients at high risk of bladder cancer would be beneficial for patients (15). While hematuria and lower urinary tract symptoms are common presenting signs of bladder cancer, the majority of patients with these symptoms do not have cancer (16, 17). Current guidelines acknowledge that urine-based tumor markers with high negative predictive values (NPVs) have the potential to further stratify risk of bladder cancer and direct prioritization of bladder cancer workup in patients most likely to have disease while reducing invasive procedures in those unlikely to have bladder cancer (18).

In the setting of bladder cancer surveillance, there is also great interest in urine-based assays to direct cystoscopy scheduling (18–20). While guidelines acknowledge the high sensitivity and NPVs of newer approved urinary biomarkers, cytology and UroVysion FISH is currently the only urine-based assay recommended by guidelines for regular use for bladder cancer surveillance (6, 7, 21). With high specificity (>90%) but low sensitivity (16%–84%) (21), a negative cytology does not negate the need for cystoscopy, limiting its utility in reducing the frequency of invasive surveillance procedures. As such, improved assays and additional validation are needed before clinical recommendation of the use of urinary biomarkers to replace or delay detection or surveillance cystoscopy (22).

To address the need for a noninvasive test with the potential to assess the need for cystoscopy, our group previously developed urine mRNA panel (uRNAp), a noninvasive urine gene expression test based on RT-qPCR of roundabout guidance receptor 1 (*ROBO1*), corticotropin releasing hormone (*CRH*), and insulin-like growth factor 2 (*IGF2*) in urine sediments, for detection and surveillance of NMIBC (22). Here, we present the results of a prospective, multicenter validation study of uRNAp for initial detection of bladder cancer and for surveillance in patients undergoing cystoscopy for a history of bladder cancer.

## Results

**Figure 1:**
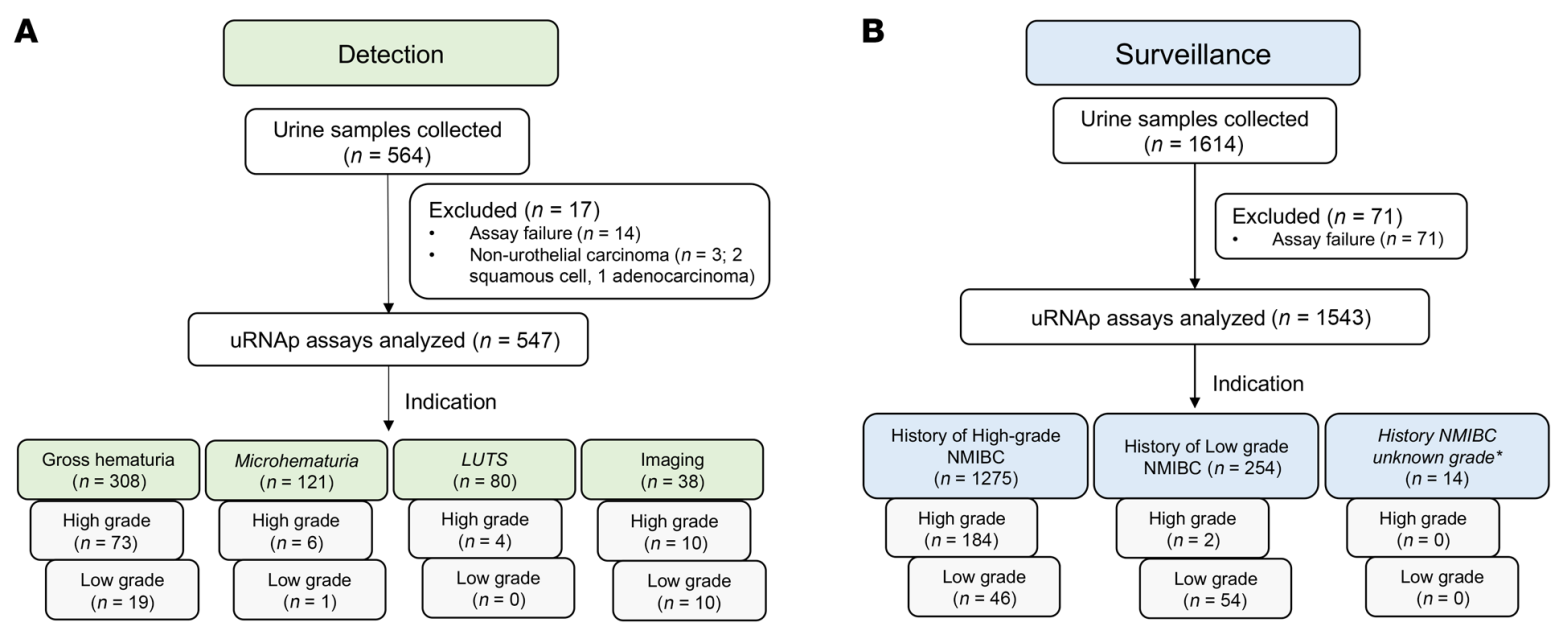
Urine samples collected and analyzed for validation of uRNAp. (**A**) For the detection cohort 547 samples were collected from 529 patients undergoing diagnostic cystoscopy for gross hematuria, microhematuria, lower urinary tract symptoms (LUTS), or incidental imaging findings (Imaging) were included in analysis. The were 123 newly diagnosed cases of bladder cancer in the detection cohort, 92 (73 high-grade [HG], 19 low-grade [LG]) from patients presenting with gross hematuria, 7 (6 HG, 1 LG) in patients with microhematuria, 4 HG in patients with LUTS, and 20 (10 HG, 10 LG) in patients with incidental imaging findings. (**B**) In the surveillance cohort 1,543 samples from 447 patients with a history of NMIBC were included for analysis. 1,275 samples were collected from patients undergoing surveillance for a history of HG NMIBC with 230 recurrences (184 HG, 46 LG). 254 samples were collected from patients with a history of LG NMIBC with 56 recurrences (2 HG, 54 LG). *Details of prior NMIBC grade were not available for 14 samples collected from 8 patients and no recurrences were found in these patients.

A total of 2,178 urine samples were prospectively collected from 856 patients across a tertiary academic medical center and a VA medical center (Tables 1 and 2). A total of 88 samples were excluded from analysis: 85 samples (4%) had insufficient cellularity (CDC42BPB cycle threshold [Ct] > 36), and 3 samples were from patients with non-urothelial carcinoma of the bladder (Figure 1). There were 547 samples from 529 patients included in the analysis for the detection cohort and 1,543 samples from 447 patients included in the analysis for the surveillance cohort (Table 1 and Figure 1). Patients in the detection cohort gave a median of 1 sample (range 1–3), while those in the surveillance cohort gave a median of 3 samples (range 1–15) at unique time points.

**Figure 2:**
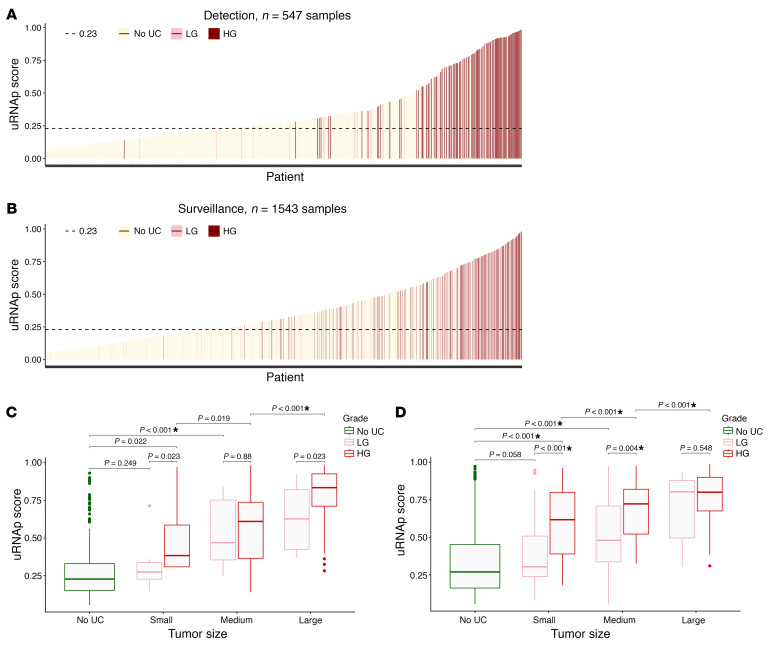
uRNAp score for UC detection and surveillance by tumor grade and aggregate size. (**A** and **B**) Waterfall plots of uRNAp scores by tumor grade from patient samples without UC (No UC, beige) and with low-grade (LG; pink) or high-grade (HG; red) UC. A uRNAp score of 0.23 (horizontal dashed line) was the cutoff for UC diagnosis in (**A**) patients without a prior history of UC (detection cohort, *n* = 547 samples) and (**B**) patients with a history of bladder cancer (surveillance cohort, *n* = 1,543 samples). (**C** and **D**) uRNAp scores increase with increasing tumor size and grade for both detection and surveillance cohorts. *P* values for ANOVA comparisons are shown above brackets. Significant *P* values that are below the Bonferroni-adjusted value for multiple comparisons of 0.002 are marked with an asterisk. (**C**) In the detection cohort 11 tumors were defined as small (<1 cm; *n* = 7 LG; *n* = 4 HG), 26 were medium (between 1 and 3 cm; *n* = 13 LG; *n* = 13 HG), and 84 were large (>3 cm; *n* = 10 LG; *n* = 74 HG). Tumor size information was not available in 2 cases. (**D**) In the surveillance cohort 82 tumors were defined as small (<1 cm; *n* = 50 LG; *n* = 32 HG), 89 were medium (between 1 and 3 cm; *n* = 33 LG; *n* = 56 HG), and 77 were large (>3 cm; *n* = 14 LG; *n* = 63 HG). Tumor size information was not available in 38 cases. Box plots show the interquartile range, median (line), and minimum and maximum (whiskers).

**Figure 3:**
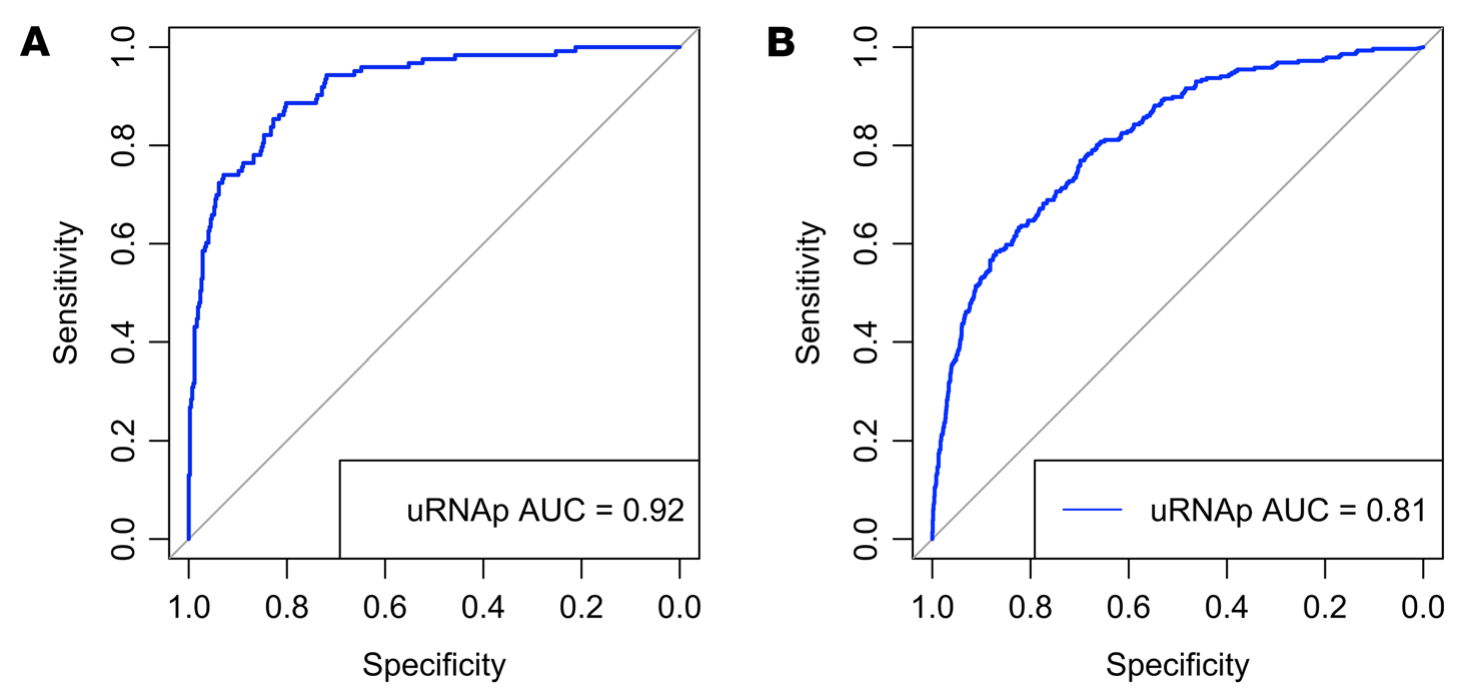
Performance of uRNAp for identification of UC. Receiver operating characteristic (ROC) curve for uRNAp in (**A**) the detection cohort of 547 samples at unique time points from 529 patients and (**B**) the surveillance cohort of 1,543 samples at unique time points from 447 patients. AUC, area under the ROC curve.

In the detection cohort, cystoscopy was performed because of gross hematuria (*n* = 308), microhematuria (*n* = 121), lower urinary tract symptoms (*n* = 80), or abnormal radiologic imaging findings (*n* = 38). In this cohort, there were 123 histologically confirmed cases of bladder cancer (30 LG, 93 HG). Using a uRNAp score cutoff of 0.23, there were 120 true positive (TP) uRNAp tests. A total of 424 samples correlated with negative cystoscopy or benign biopsy, of which 215 had true negative (TN) uRNAp tests. The distribution of uRNAp scores for patients in the detection cohort is shown in Figure 2A. For those patients without a prior history of bladder cancer, uRNAp demonstrated 98% sensitivity, 51% specificity, 96% NPV, and 36% positive predictive value (PPV). Clinical cytology was available for 63% (*n* = 345) of the patients in the detection cohort. As expected, cytology had high specificity (99%) with lower sensitivity of 33% overall and 48% for detection of HG bladder cancer (Table 3). In all cases where cytology was positive, the uRNAp score was also positive. Receiver operating characteristic (ROC) analysis of uRNAp in the detection cohort demonstrated an area under the curve (AUC) of 0.92 (Figure 3A). While the detection cohort included all patients undergoing cystoscopy for signs and symptoms of potential bladder cancer, the majority of patients were being evaluated because of hematuria (gross hematuria *n* = 308, 92 incidences of bladder cancer; microhematuria *n* = 121, 7 incidences of bladder cancer). Of the 121 patients with microhematuria, 93 were considered at high risk for bladder cancer, 19 at intermediate risk, and 9 at low risk based on American Urological Association (AUA) microhematuria guidelines (16, 17). For high-risk hematuria patients uRNAp demonstrated 98% sensitivity, 50% specificity, and 99% NPV. No cancers were found in patients undergoing cystoscopy for intermediate- or low-risk hematuria. In these patients uRNAp specificity was 79% for intermediate-risk hematuria (*n* = 19) and 56% for low-risk hematuria (*n* = 9).

In patients with a prior history of bladder cancer (surveillance cohort), there were 286 histologically confirmed recurrences (100 LG, 186 HG). This cohort included 1,257 samples from negative cystoscopy or benign biopsy with 537 TN uRNAp tests and 286 incidences of bladder cancer with 268 TP uRNAp results. Overall, uRNAp demonstrated 94% sensitivity, 43% specificity, 97% NPV, and 27% PPV in the surveillance cohort (Table 3). For HG recurrence uRNAp sensitivity was 99%, and for LG recurrences the sensitivity was 86%. Distribution of uRNAp scores for the surveillance cohort is shown in Figure 2B. For the surveillance population 83% of the patients had corresponding urine cytology that had an overall sensitivity of 33% and specificity of 99% for identification of recurrent disease and 49% sensitivity for HG recurrence. ROC analysis of uRNAp in the surveillance population demonstrated an AUC of 0.81 (Figure 3B). There was no difference in model performance between medical centers for either the detection or surveillance populations.

Thirteen patients undergoing surveillance for a history of bladder cancer were found to have pathologically confirmed upper tract UC (UTUC) with no evidence of disease in the bladder. Investigation of the upper tract was initiated for 7 of these patients due to positive cytology with negative cystoscopy. Three patients had new onset of gross hematuria with negative cystoscopy, and subsequent imaging indicated upper tract disease. Two patients underwent imaging for other symptoms that revealed potential upper tract tumors. In 1 patient, a tumor emanating from the ureter was seen on cystoscopy. Four of the UTUCs were LG and 9 were HG. In all cases the uRNAp score predicted the presence of UTUC.

In both the detection and surveillance cohorts, we found that HG tumors had significantly higher uRNAp scores for comparably sized LG tumors (*P* < 0.05). Tumor size was reported in the clinical records for 98% of the patients with bladder cancer in the detection cohort (*n* = 121) and 87% of the patients in the surveillance cohort (*n* = 248). Figure 2, C and D, show that uRNAp score significantly increased with both tumor size and grade. Large (>3 cm) HG tumors had the highest median uRNAp score of 0.8340 in the detection cohort and 0.8004 in the surveillance cohort, while small (<1 cm) LG tumors had the lowest scores (median 0.2745 detection and 0.3036 surveillance). Consistent with this finding, the majority of false negative uRNAp scores observed were from patients with small LG tumors (12 of 18).

While grade and size are likely the primary factors influencing uRNAp scores, increased uRNAp scores were found with higher stage disease (Supplemental Figure 1; supplemental material available online with this article; https://doi.org/10.1172/JCI203094DS1). Ta tumors had the lowest uRNAp scores (0.5563), followed by Tis (0.6958). Invasive tumors had significantly higher uRNAp scores than noninvasive tumor, but there was no difference between T1 (0.7651) and T2 (0.7556). If we separated LG Ta and HG Ta tumors, we observed 4 significantly different populations: no bladder cancer (uRNAp average 0.3065), LG Ta (uRNAp average 0.4817), HG Ta and Tis (uRNAp average 0.6626), and HG T1 and T2 (uRNAp average 0.7617).

UC histologic subtypes were reported by pathology for 16 patients in the detection cohort and 12 patients in the surveillance cohort. Subtypes included squamous differentiation, micropapillary, plasmacytoid, neuroendocrine, small cell carcinoma, and poorly differentiated (Supporting Data Values). Consistent with the performance for HG disease, uRNAp predicted bladder cancer in all cases with histologic subtypes with a median uRNAp score for patients with variant histology of 0.7829 in the detection cohort and 0.8187 in the surveillance cohort.

While the positivity threshold of 0.23 was set to maximize sensitivity, setting a high specificity threshold may be useful to identify high-risk patients for expedited cystoscopy. In the detection cohort, a threshold of 0.62 yielded 95% specificity with a PPV of 0.802. In the surveillance cohort a threshold of 0.75 gave 95% specificity with a PPV of 0.63.

Given the overlapping patient demographics, we investigated prostate cancer as a potential confounder and found no discernable effect on uRNAp assay performance. In the detection cohort, 78 samples from 76 men with a history of prostate cancer were assayed. All new incidences of bladder cancer (*n* = 16, 13 HG, 3 LG) in men with a history of prostate cancer were positively detected by uRNAp with specificity of 54%. In the surveillance cohort, 161 samples from 48 men with a history of prostate cancer were assayed. Twenty-three of the 25 recurrences (13 HG, 12 LG in men with a history of prostate cancer) were detected, yielding a sensitivity of 92% with specificity of 43%.

**Figure 4:**
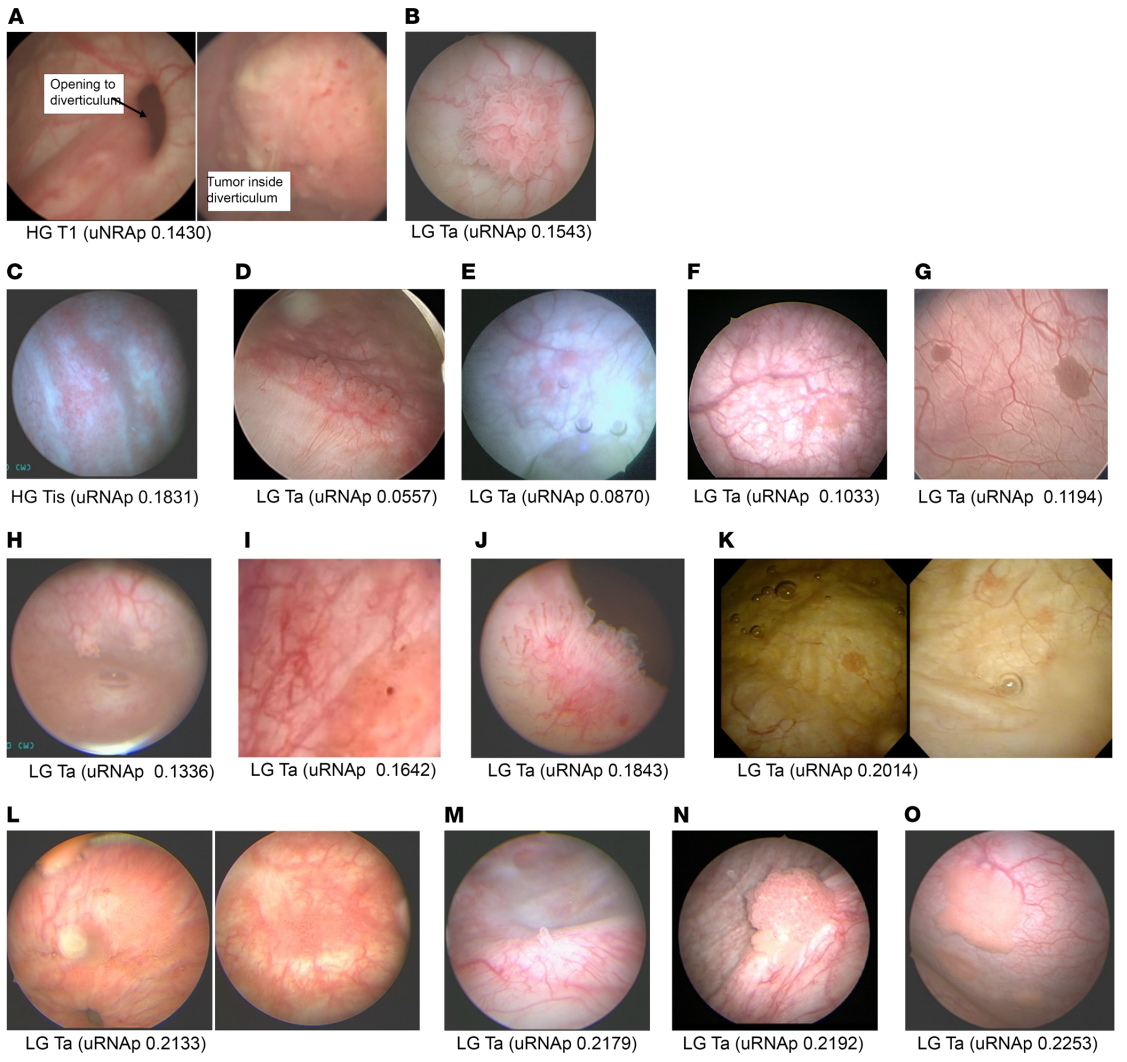
Cystoscopic appearance of bladder cancer cases with false negative uRNAp test. Images taken during tumor resection or clinic cystoscopy were available for 2 of 3 uRNAp false negatives in the detection cohort (**A** and **B**) and 13 of 18 uRNAp false negatives in the surveillance cohort (**C**–**O**). In the detection cohort, the 1 HG tumor with a false negative uRNAp score was inside a diverticulum (**A**). The 2 LG tumors with false negative uRNAp scores were small (<1 cm) solitary tumors (**B**, and no image available, uRNAp = 0.2191). Cytology was also negative in all 3 of these uRNAp false negative cases. In the surveillance cohort (**C**–**O**) only 1 HG recurrence was missed by the uRNAp assay: a 5–10 mm patch of CIS (**C**). The remainder of the uRNAp false negatives were from LG recurrences. 12 were small (<1 cm aggregate tumor, **E**–**K** and **M**, and no image available, *n* = 4 uRNAp scores 0.1015, 0.1214, 0.1372, 0.1874) and 5 medium (1–3 cm aggregate tumor size, **D**, **L**, **N**, and **O**, and no image available, *n* = 1 uRNAp score 0.2158).

To better understand the assay performance, we further examined the false negative uRNAp assays (Figure 4). Based on clinical descriptions, available intraoperative photographs, and pathology, we found that 2 of the 3 false negative uRNAp assays in the detection cohort were small (<1 cm) LG tumors. The 1 HG tumor with a false negative uRNAp assay in the detection cohort was a 2 cm T1 tumor in a bladder diverticulum with a narrow opening (Figure 4A). In the surveillance cohort 12 of the 18 false negative uRNAp assays were small (<1 cm aggregate tumor size) LG Ta recurrences. Five of the uRNAp false negatives were LG Ta recurrences that fell in the medium size category (1–3 cm aggregate tumor size). The 1 HG false negative from the surveillance cohort was a small (~1 cm) patch of CIS. Notably, the 3 incidences of uRNAp false negative in the detection cohort also had negative cytology. Thirteen of the 18 false negative uRNAp cases in the surveillance cohort had corresponding cytology. However, cytology was also negative in these cases.

## Discussion

We have validated uRNAp, a simple, noninvasive test based on 3 urinary mRNAs for detection and surveillance of bladder cancer in a prospective observational study with over 2,000 urine samples from 976 patients at 2 health care systems. In patients without history of bladder cancer undergoing urologic evaluation for hematuria and other lower urinary tract symptoms (detection cohort), uRNAp demonstrated 98% sensitivity, 51% specificity, and NPV of 99%. In patients with known history of bladder cancer (surveillance cohort), uRNAp demonstrated 94% sensitivity, 43% specificity, and NPV of 97%. Notably, the sensitivity for HG recurrence in the surveillance cohort was 99%.

The high sensitivity and high NPV of the uRNAp support the potential use of uRNAp as a tool to optimize the timing of cystoscopies for bladder cancer, particularly in de-escalation of the invasive cystoscopy in patients at low risk of disease. AUA guidelines recommend a bladder cancer workup for patients with hematuria based on risk stratification factors including age, sex, smoking history, and the degree of hematuria (5, 23). The diagnostic yield for bladder cancer in patients with microhematuria is notably low, and guidelines recommend screening de-escalation for individuals without known risk factors for bladder cancer (16). Adding a simple urine-based assay with performance characteristics of uRNAp could add an objective measure to assess the immediacy of the need for cystoscopy in low-risk patients. Patients with gross hematuria are considered at high risk for bladder cancer, and recommendations are for a computed tomography urogram and cystoscopy. We do not foresee uRNAp replacing the recommended initial workup for high-risk patients, but the assay could serve as an adjunct diagnostic test. Notably, the 3 false negative uRNAp results in the detection cohort were from patients considered high-risk based on degree of hematuria, age, and smoking history. Conversely, for patients without a UC diagnosis who receive multiple cystoscopies due to episodic gross hematuria or persistent microhematuria, uRNAp could be used for monitoring the likelihood of urothelial cancer occurrence, thus providing a good alternative to repeat cystoscopies. For intermediate- and high-risk patients based on age and smoking/exposure history with microhematuria, uRNAp could provide a useful adjunct to determine the urgency of cystoscopy, especially for patients without easy access to specialists.

The 3 mRNAs in the uRNAp assay, *ROBO1*, *IGF2*, and *CRH*, were chosen for expression in urine of patients with bladder cancer, and they have been associated with bladder cancer (24–28). *ROBO1* encodes a transmembrane protein and receptor for the secreted glycoprotein SLIT. Signaling through ROBO1 plays a role in tumor angiogenesis and metastasis in a variety of cancers (24, 25, 29). *IGF2* expression is upregulated in many bladder tumors and may play a role in tumor cell proliferation (27, 30). *CRH* encodes a secreted peptide hormone involved in stress response (31). CRH may function in bladder cancer signaling promoting cell migration (30).

Patients with a diagnosis of NMIBC are subjected to surveillance cystoscopies up to every 3 months. Taking advantage of the high NPV of uRNAp, it could be used to monitor recurrence and reduce the frequency of cystoscopy, particularly in patients with low- and intermediate-risk bladder cancer. For example, 1 study participant was initially diagnosed with low-risk bladder cancer (solitary LG Ta tumor) and had recurrence with multifocal LG Ta 2 years later, then was followed by surveillance cystoscopy without recurrence. The uRNAp assay was positive at the initial diagnosis and recurrence and appropriately negative at all follow-up procedures. Another patient had an initial diagnosis of HG Ta and an HG Ta recurrence 1 year later. The patient was then treated with induction Bacillus Calmette-Guérin (BCG) and has remained bladder cancer recurrence free since. The uRNAp assay was positive at the initial diagnosis and recurrence, and 5 of 7 negative surveillance cystoscopies could have been avoided based on the uRNAp assay. Conversely, a persistently positive uRNAp may indicate undetected disease and warrant repeat diagnostic workup and close surveillance. For example, 1 patient with a long history of recurrent high-risk NMIBC and several treatments, including intravesical BCG and mitomycin and systemic pembrolizumab, had persistently elevated uRNAp scores over a 12-month period without detectable recurrence. Eventually, a positive cytology prompted a biopsy that found CIS.

Higher uRNAp scores are strongly correlated with high-risk disease, though there was a subset of false positives with uRNAp greater than the threshold for 95% specificity. Two patients in the detection cohort with high uRNAp scores were diagnosed with bladder cancer less than 1 year after a high false negative uRNAp test. For example, 1 of the detection patients had a uRNAp score of 0.9301 at cystoscopy and suspicious cytology, followed by a bladder biopsy of an erythematous lesion that was pathologically determined to be benign inflammation. While this sample was identified as a false positive uRNAp assay, this patient returned for a second biopsy 8 months later that found HG T1 bladder cancer, suggesting that the high uRNAp score may have been due to bladder cancer that initially went undetected. In the surveillance cohort, 3 patients had suspicious cytology and a uRNAp score > 0.75 (uRNAp: 0.9101, 0.9354, and 0.8429). While the associated biopsy was benign, each of these patients were found to have CIS at a later cystoscopy. One surveillance patient accounted for 4 false negative uRNAp results above the high specificity threshold (uRNAp: 0.8266, 0.8620, 0.8360, and 0.7696) from samples collected 3 months apart. Cystoscopy notes for this patient described a stable, benign-appearing nodule at the bladder neck. After over a year of surveillance, the patient had positive cytology, and biopsy of this lesion found HG T1 disease. However, some benign lesions may give high false positive results. For example, a surveillance patient with a history of HG bladder cancer had a surveillance uRNAp assay of 0.9726 and a papillary lesion found on cystoscopy. However, on biopsy the lesion was found to be nephrogenic adenoma, a benign lesion.

Given that 17 of the 18 uRNAp false negatives in the surveillance cohort were LG Ta tumors, the risk of delayed recognition of recurrence may be acceptable to practitioners and patients. Several studies have looked at the safety of management of LG tumors with active surveillance to reduce the morbidity and cost of frequent TURBT (32–34). These studies found that LG tumors rarely progress and that small LG tumors in asymptomatic patients and do not necessitate immediate TURBT. Urine biomarkers could play a key role in active surveillance and de-intensification of bladder cancer management (19, 32, 34).

The single HG T1 tumor that was a uRNAp false negative provided insight into a potential limitation of urinary biomarkers. This tumor was completely contained within a bladder diverticulum, with no evidence of disease elsewhere in the urinary tract. A second urine sample collected from this patient at the time of TURBT had a positive, albeit relatively low, uRNAp (0.3433) for a 2 cm HG tumor. We speculate that diverticulum shielded the tumor from exfoliating tumor cells into the urine (35). Bladder diverticula are typically seen on a cross-sectional imaging that patients undergo at initial workup for suspected bladder cancer and might indicate a need to proceed with caution when considering the use of urine biomarkers. Clinical use of this and other urine-based assays should consider how anatomical abnormalities might affect the tests.

Cytology and adjuncts to cytology, such as UroVysion FISH, are currently the only urine assay recommended as part of clinical use for bladder cancer (7, 21). However, several promising urinary biomarker assays targeting different components of urine (e.g., proteins, DNA, RNA) are commercially available and are starting to be recognized to have potential in clinical guidelines (15, 36, 37). The CxBladder assays (Triage/Detect and Monitor, Pacific Edge) and Xpert Bladder (Cepheid) are the most closely related to uRNAp. Both assays target mRNA in exfoliated urine cells for gene expression analysis, and both have high sensitivity and high NPVs for bladder cancer (18–20). Recently, studies evaluating these commercially available urine gene expression assays to triage patients with microhematuria (38) (CxBladder Triage) or guide surveillance cystoscopy timing in low-risk (19, 20) (CxBladder Monitor) or high-risk (18) (Xpert Bladder Monitor) NMIBC found the test could be safely implemented into clinical practice, and study patients had high satisfaction scores (19).

While overall performance characteristics of uRNAp and the Xpert Bladder and CxBladder assays are comparable, the genes assayed in the tests are different (15). We note that uRNAp is currently not a Clinical Laboratory Improvement Amendments–certified (CLIA-certified) assay. Due to the heterogeneity of bladder cancer, having multiple assays may prove beneficial, and one test may perform better in some patients or populations. Additionally, these assays may not be available to all patients; for example, Xpert Bladder assays are not available in the United States. Ongoing work is in progress to develop uRNAp as a CLIA-certified assay that could be performed in any clinical laboratory. This may be especially useful in settings such as the VA health care system, where the patient population is at high risk for bladder cancer and resources are limited (39).

Our validation of uRNAp for surveillance focused on patients with a history of bladder cancer. However, patients with a history of bladder cancer are at increased risk for tumors in the upper urinary tract. Therefore, an assay that is sensitive for detection of UTUC would be a welcome clinical adjunct for clinicians. As has been found in similar biomarker assays (Xpert Bladder and CxBladder) (18, 19), all instances of UTUC in patients undergoing surveillance for bladder cancer were identified with the uRNAp assay. A targeted cohort of patients with UTUC is needed to accurately assess uRNAp performance for UTUC.

The observational nature of this study is a limiting factor, as patient and provider acceptance of the assay are key factors in assessing clinical utility. However, this validation study provides the foundation for a well-designed randomized controlled trial needed to incorporate uRNAp in clinical practice. An initial clinical trial would likely focus on the use of uRNAp to rule out the need and/or reduce the frequency for surveillance cystoscopy in patients with low-risk bladder cancer. To provide the quality control standards necessary for a clinical trial, the assay will need to be moved from the research lab to a CLIA-regulated clinical laboratory to facilitate clinical translation (40). Since the uRNAp assay uses a standard real-time PCR approach, this aspect of translation is feasible. While the current study collected samples from 2 distinct sites, testing in a large multicenter trial will further broaden the patient diversity to assess uRNAp performance. In this study we had a small cohort of patients with multiple samples assayed over their course of treatment. As bladder cancer patients undergo frequent cystoscopies, understanding the longitudinal change in uRNAp in a larger patient cohort will provide additional insight into the prognostic utility of uRNAp.

Prospective validation of uRNAp strongly supports its promising clinical utility as an adjunct to cystoscopy and ureteroscopy for diagnosis and management of UC. While the specificity was lower in this independent validation study compared with our initial report (22), the high sensitivity and NPVs are indicative of the translational potential. Additional clinical assessment will determine the potential of uRNAp to allow for fewer cystoscopic procedures and improve patient care and quality of life.

## Methods

### Sex as a biological variable.

Sex was not considered as a biological variable in this study. Male and female participants were included. However, as bladder cancer disproportionately affects men (~4:1) compared with women and the recruitment took place at a VA medical center, the majority of the patients were men.

### Patient population.

In our observational study, adult patients undergoing diagnostic cystoscopy for signs or symptoms of bladder cancer or surveillance of bladder cancer at Veterans Affairs Palo Alto Health Care System (VAPAHCS) and Stanford Health Care (SHC) between May 2019 to October 2025 were study eligible. Exclusion criteria included a history of muscle-invasive bladder cancer or muscle-invasive UTUC. There was no attrition, as provision of follow-up samples was not required for study participation. Demographic data and risk stratification factors including age, sex, smoking history, and the degree of hematuria were collected for all participants (Table 1). The detection cohort included patients without a prior diagnosis of bladder cancer undergoing cystoscopy for gross hematuria, microhematuria, lower urinary tract symptoms, or abnormal findings on radiologic imaging. The surveillance cohort included patients with a history of NMIBC undergoing surveillance cystoscopy. Tumor size was defined based on a combination of radiographic imaging studies (if available) and as estimated by the experienced surgeon and reflected in operative reports. Small tumors are defined as <1 cm; medium, 1–3 cm; and large, >3 cm. Images of tumors were taken at the discretion of the surgeon and obtained from patient medical records. Risk categorization was based on AUA guidelines (7, 23).

A minimum sample of 10 mL urine was collected from participants at scheduled clinical encounters as part of standard clinical care. For patients undergoing cystoscopy, voided urine was collected prior to the procedure. If patients were unable to void, urine collection was performed via the cystoscope upon insertion. For patients providing multiple samples included in analysis, samples were collected at unique appointments at least 3 months apart. Visual assessment by cystoscopy was required for inclusion in statistical analysis. Patients with visualized lesions suspicious for malignancy or indeterminant regions of interest on cystoscopy underwent TURBT, and patients with concern for possible upper urinary tract tumor underwent ureteroscopy. Cancer diagnosis was based on histopathology of biopsy samples. For patients who provided samples prior to both clinic cystoscopy and subsequent biopsy, the urine sample from the cystoscopy was used in the analysis, as it more closely mirrors the potential clinical application for uRNAp. Treating clinicians were blinded to uRNAp assay data prior to procedures.

### Sample preparation and analysis.

Ten milliliters of urine was separated by centrifugation for 10 minutes at 1,000*g* to collect the sediment for RNA isolation and uRNAp assay. RNA was isolated from the sediment using Zymo Research Direct-zol RNA isolation kit (catalog R2062) or QIAGEN RNeasy (catalog 74106). No substantial difference was found with different methods of RNA preparation. RT-qPCR was performed to evaluate expression of *ROBO1*, *CRH*, *IGF2*, and *CDC42BPB*, a housekeeping gene to ensure adequate sample cellularity, as previously described (22). RT reactions were performed using High-Capacity RNA-to-cDNA Kit (Thermo Fisher Scientific, 4388950) and qPCR with TaqMan Fast Advanced Master Mix (Thermo Fisher Scientific catalog 4444964) and TaqMan probes (Thermo Fisher Scientific, Assay IDs Hs00178787_m1, Hs00268049_m1, Hs00174941_m1, Hs01005963_m1) and analyzed on a QuantStudio 5 (Thermo Fisher Scientific). Ct values for gene expression were determined, with a Ct of 41 assigned for undetermined Ct values. *ROBO1*, *CRH*, and *IGF2* were used to calculate the uRNAp score using the following equation: *P* = exp[*A*]/(1 + exp[*A*]), where *A* = −0.1857 − 0.20551 × *ROBO1* Ct − 0.71388 × *CRH* Ct − 0.98452 × *IGF2* Ct.

*CDC42BPB* served as a sample adequacy control to ensure the sample input contained enough cells for reliable assay performance. Samples with *CDC42BPB* Ct values > 36 were excluded due to low cellularity.

### Statistics.

Statistical analyses were performed in accordance with established guidelines (41). Of the samples collected, 547 samples in the detection cohort and 1,543 in the surveillance cohort were suitable for analysis (Figure 1). With 123 incidences of bladder cancer in the detection cohort and 286 incidences in the surveillance cohort, the study exceeded the criterion of 30 events per variable for prediction model development using 3 genomic predictors (42). All samples included in the study are described at both patient and sample level (Tables 1 and 2) using mean with IQR for continuous characters, and percentage for categorical characters. A uRNAp score of each sample was calculated based on previously published methods (22). Due to a change in RT-qPCR methodology from the prior report, an adjustment was made using retrospective data to the coefficients and intercept in the regression model. From the modified model, a cutoff of 0.23 was established as the threshold for test positivity as described previously (22). The primary outcome was the presence of pathologically confirmed UC of the bladder. Patients with adenocarcinoma or squamous cell carcinoma of the bladder were excluded.

The performance of uRNAp to detect the presence of pathologically confirmed bladder cancer on cystoscopy was assessed using ROC using AUC, sensitivity, specificity, PPV, and NPV. All analyses were performed on detection and surveillance cohorts separately (43).

R studio (version 2024.04.2, https://www.r-project.org) was used to perform all the statistical analyses, including key packages pROC, dcurves, and ggplot2. Two-way ANOVA test was used for the comparisons on uRNAp scores across tumor sizes with significance level of *P* < 0.05 for a 2-sided test. Study data and presentation were assessed for rigor using STROBE criteria (44). The uRNAp data generated in this study are available in Supporting Data Values.

### Study approval.

The study protocol was approved by Stanford University Institutional Review Board (protocol 55427) and VAPAHCS Research and Development Committee (protocol LIA0028). Written informed consent was obtained from participants prior to sample collection.

### Data availability.

Supporting data values including biomarker Ct values and calculated uRNAp scores available in Supporting Data Values XLS file.

## Author contributions

KEM, ZK, ES, and JCL designed the study. KEM, IY, and ZK conducted the experiments. VL, TJL, and GR obtained informed consent from study participants and coordinated sample collection and transport. ES, AET, KBP, JBS, JTL, and ECS provided reagents and samples. KEM, ZK, JL, and JCL analyzed data. JL provided statistical analysis. KEM and JCL wrote the manuscript.

## Conflict of interest

The authors have declared that no conflict of interest exists.

## Funding support

- Department of Veterans Affairs Merit Review I01 BX005598 (to JCL).

## Supplementary Material

Supplemental data

ICMJE disclosure forms

Supporting data values

## Figures and Tables

**Table 1:**
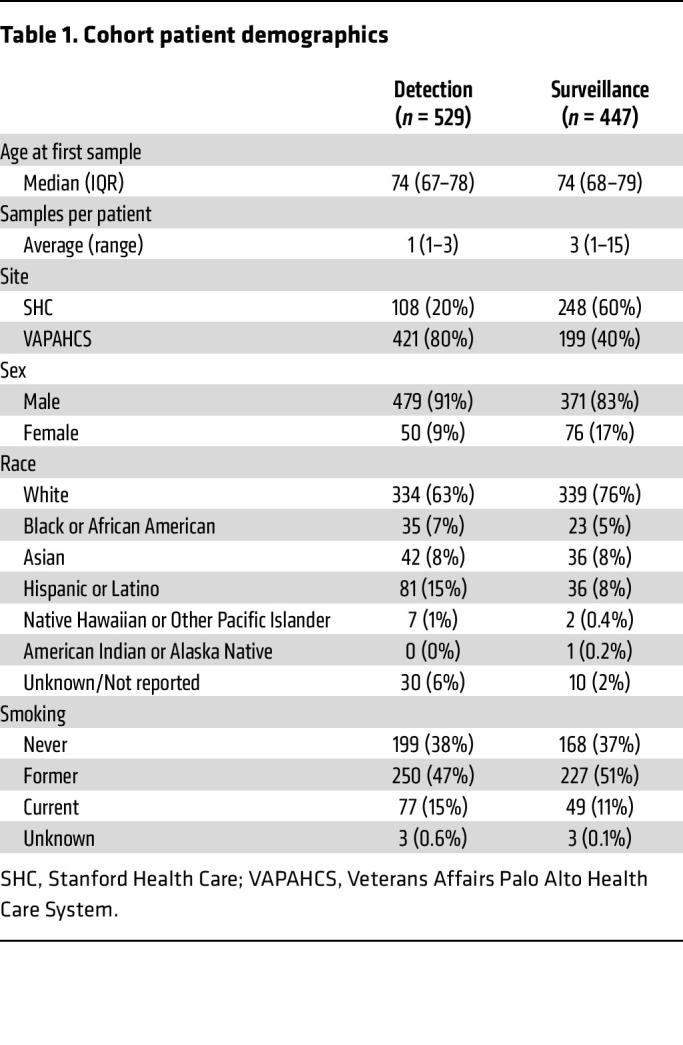
Cohort patient demographics

**Table 2:**
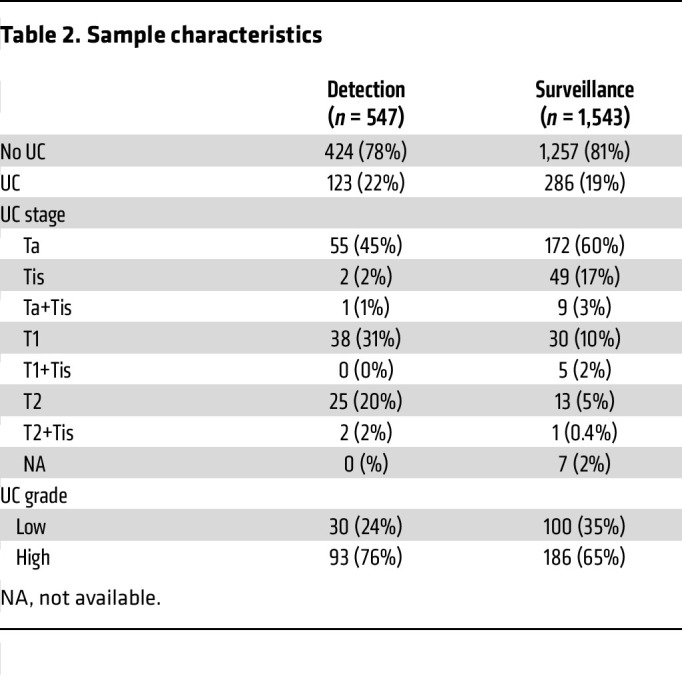
Sample characteristics

**Table 3:**
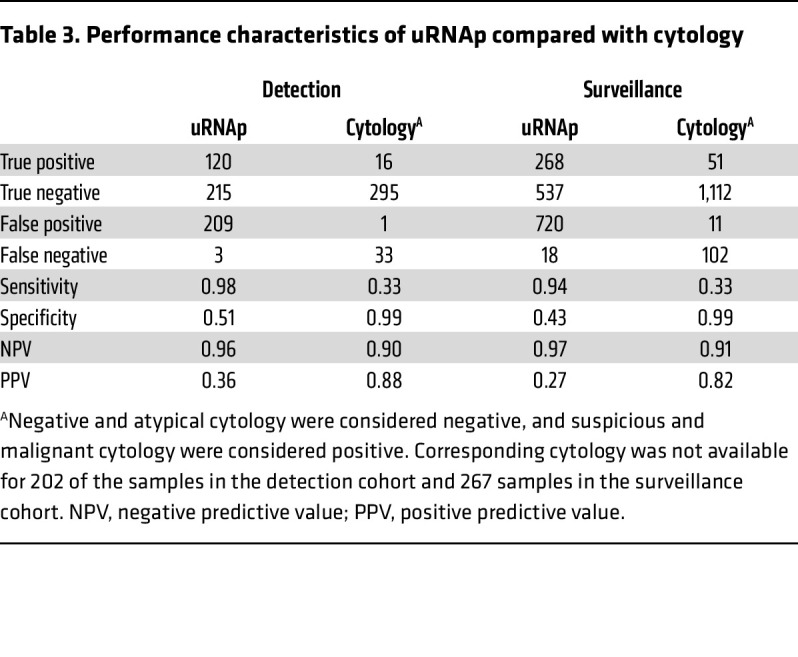
Performance characteristics of uRNAp compared with cytology

## Version 1 04/09/2026

In-Press Preview

## Version 2 06/01/2026

Electronic publication
