# Supplementary material for: Large multicenter validation of urine RNA profile for urothelial carcinoma detection and surveillance
Source: J Clin Invest. 2026 Apr 9;136(11):e203094. doi: 10.1172/JCI203094 (PMC13221218; doi:10.1172/JCI203094)
Supplement: Supplemental data [file jci-136-203094-s177.pdf]

Supplementary figure 1

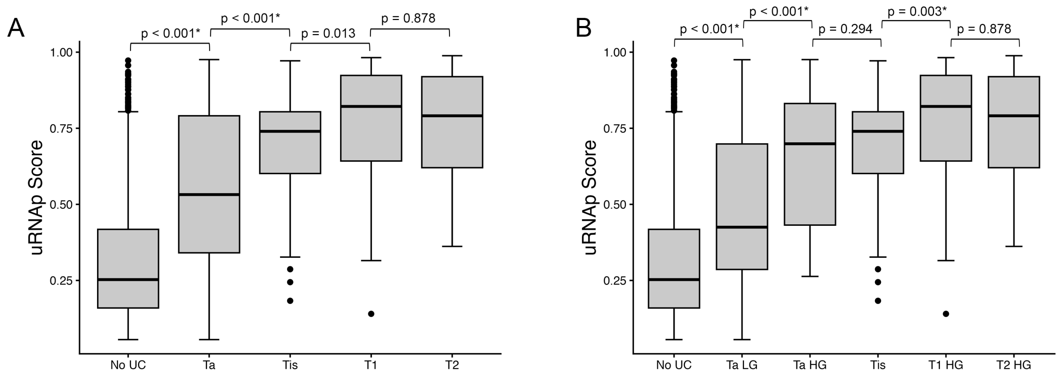

Figure S1 Urine RNA profile (uRNAp) score by tumor stage. Overall, uRNAp scores were higher with higher stage. (**A**) Ta tumors had significantly higher average uRNAp scores than samples without urothelial cancer (No UC 0.3065; Ta 0.5563), and Tis tumors were significantly higher than Ta (Tis 0.6958) but significantly lower than invasive T1 and T2 tumors (T1 0.7651; T2 0.7556). There was no significant difference between T1 and T2 tumors. (**B**) There were four significantly different populations: no bladder cancer (uRNAp average 0.3065); LG Ta (uRNAp average 0.4817); HG Ta and Tis (uRNAp average 0.6626); and HG T1 and T2 (uRNAp average 0.7617). Brackets indicate ANOVA comparisons, p values < 0.01 are significant.
